# Crystal structures and molecular dynamics simulations of a humanised antibody fragment at acidic to basic pH

**DOI:** 10.1038/s41598-023-42698-7

**Published:** 2023-09-28

**Authors:** Jiazhi Tang, Cheng Zhang, Nuria Codina Castillo, Christophe J. Lalaurie, Xin Gao, Paul A. Dalby, Frank Kozielski

**Affiliations:** 1 UCL School of Pharmacy, 29-39 Brunswick Square, London, WC1N 1AX UK; 2 Department of Biochemical Engineering, UCL, Bernard Katz Building, Gower Street, London, WC1E 6BT UK; 3 Division of Biosciences, Department of Structural and Molecular Biology, UCL, London, WC1E 6BT UK

**Keywords:** Computational biophysics, Molecular modelling, SAXS, X-ray crystallography

## Abstract

Antibody-fragment (Fab) therapy development has the potential to be accelerated by computational modelling and simulations that predict their target binding, stability, formulation, manufacturability, and the impact of further protein engineering. Such approaches are currently predicated on starting with good crystal structures that closely represent those found under the solution conditions to be simulated. A33 Fab, is an undeveloped immunotherapeutic antibody candidate that was targeted to the human A33 antigen homogeneously expressed in 95% cases of primary and metastatic colorectal cancers. It is now used as a very well characterised testing ground for developing analytics, formulation and protein engineering strategies, and to gain a deeper understanding of mechanisms of destabilisation, representative of the wider therapeutic Fab platform. In this article, we report the structure of A33 Fab in two different crystal forms obtained at acidic and basic pH. The structures overlapped with RMSD of 1.33 Å overall, yet only 0.5 Å and 0.76 Å for the variable- and constant regions alone. While most of the differences were within experimental error, the switch linker between the variable and the constant regions showed some small differences between the two pHs. The two structures then enabled a direct evaluation of the impact of initial crystal structure selection on the outcomes of molecular dynamics simulations under different conditions, and their subsequent use for determining best fit solution structures using previously obtained small-angle x-ray scattering (SAXS) data. The differences in the two structures did not have a major impact on MD simulations regardless of the pH, other than a slight persistence of structure affecting the solvent accessibility of one of the predicted APR regions of A33 Fab. Interestingly, despite being obtained at pH 4 and pH 9, the two crystal structures were more similar to the SAXS solution structures obtained at pH 7, than to those at pH 4 or pH 9. Furthermore, the *P*6_5_ crystal structure from pH 4 was also a better representation of the solution structures at any other pH, than was the *P*1 structure obtained at pH 9. Thus, while obtained at different pH, the two crystal structures may represent highly (*P*6_5_) and lesser (*P*1) populated species that both exist at pH 7 in solution. These results now lay the foundation for confident MD simulations of A33 Fab that rationalise or predict behaviours in a range of conditions.

## Introduction

A33 Fab, is an undeveloped immunotherapeutic fully-humanised antibody candidate that was targeted to the human A33 antigen^1^. The fully-humanized A33 Fab was developed by grafting the CDRs from a previous mAb candidate, into the variable region framework of a human antibody^2^. A33 antigen is expressed in the epithelia of the lower gastrointestinal tract, as well as carcinoma lesions that originate from the rectal and colonic mucosa^3^. The A33 antigen expressed in metastatic colorectal cancers share 95% similarity^4^. Despite not progressing to a commercial release, A33 Fab is a highly characterised representative of the wider therapeutic Fab platform, due to its use as a testing ground for the development of analytics^5^, formulation strategies^6–8^, protein engineering tools^9^, and to gain a deeper understanding of destabilising mechanisms^10–12^. All of these studies have used the H/C226S variant which mutated cystein 226 in the heavy chain to serine, to eliminate intermolecular dimerisation^10^, and will be referred to simply as A33 Fab throughout.

As the conformational flexibility and kinetic stability of A33 Fab have become better understood through combined biophysical and computational approaches^9,10^, and as a wide range of formulations (pH and ionic strength) and mutations have been experimentally characterised, the potential to use computational approaches such as all-atom molecular dynamics (MD) simulations with greater precision in the prediction of protein formulation behaviour has arisen. While small-angle X-ray scattering experiments, and initial MD simulations indicate important structural changes as a function of pH, the MD simulations have only been carried out using homology model structures as starting points which do not take into account any differences in structure induced by the pH. While those studies have been informative in helping to explain experimentally observed behaviours, the confidence with which such simulations can be used to predict new behaviours under different conditions or upon mutation, may depend on the accuracy of the starting structures and conditions used, and how well they represent the actual structure in those different conditions^13^. There are currently no crystal structures of A33 Fab under any conditions, or for any of the A33 Fab variants characterised experimentally. Therefore, to investigate whether the impact of pH on A33 Fab structure can be captured during crystallography, we obtained crystal structures at two different pH.

We crystallized the A33 Fab at both pH 9 and pH 4, which gave rise to structures from two different space groups, *P*1 and *P*6_5_, that were solved to 2.5 and 2.2 Å resolution, respectively. These provide insights into the general structure of the A33 Fab as well as the six CDRs that originated from the A33 mAb. Initially, this was compared with structures of Certolizumab, a humanised therapeutic antibody developed from the same humanization framework but targeting a different antigen and elucidated the differences in their CDR- and switch regions. This ruled out any similarities between the Fab elbow angles observed between the two structures of A33 Fab, and those observed for Certolizumab when bound or unbound to antigen.

We then used the two crystal structures to explore the importance of differences in starting structure on the outcomes of MD simulations carried out at various pH. MD simulations starting from each of the crystal structures, evaluated the influence of starting structure on the simulations, and explored the dynamics and range of conformations accessible to A33 Fab at the respective crystallisation and neutral pH. These were further compared by using MD simulations to fit against experimental SAXS data obtained previously at various pH. Together, these showed that the simulation pH had a far greater influence on MD trajectories than did the starting structure. It also revealed that both of the crystal structures were highly representative of SAXS solution structures at pH 7, despite having been obtained in crystallisation conditions of pH 4 and pH 9.

## Materials and methods

### A33 Fab sample preparation

Expression, purification and buffer exchange of A33 Fab has been described previously^9^. Purified A33 Fab was further concentrated to 22.2 mg/ml in water with a 30 kDa Vivaspins (Generon Ltd., Bershire, UK). Prior to crystallization, samples were diluted with PIPES buffer (10 × stock of 50 mM PIPES pH 7.0 and 100 mM NaCl) and MilliQ water to a final concentration of 10 mg/ml, 15 mg/ml and 20 mg/ml, respectively.

### Protein crystallization

Purified A33 Fab was used to screen for crystallization conditions using the Low Ionic Strength Screen Kit (Hampton Research). Hanging drops were set up in 24-well Linbro plates (Molecular Dimensions) with 4 μl protein solution, 2 μl buffer reagent and 5 μl precipitant reagent, which were equilibrated against 1 mL of 24% w/v PEG3350 reservoir as described in the Low Ionic Strength Screen handbook (Hampton Research). Drops were set up at both 277 K and 291 K.

Crystals grew in two different space groups. Triclinic crystals (Space group *P1*, 2.5 Å resolution) grew from 4 μl protein solution at 20 mg/ml mixed with 2 μl 50 mM Glycine pH 9.0 and 5 μl 16% w/v PEG3350 at 291 K. The crystals were cryoprotected with 60 mM Glycine pH 9.0, 19% PEG3350 and 15% Glycerol and flash-frozen in liquid nitrogen. Hexagonal crystals (Space group *P*6_5_, 2.2 Å resolution) grew from 4 μl protein solution at 20 mg/ml mixed with 2 μl 50 mM Citric Acid pH 4.0 and 5 μl 8% w/v PEG3350 at 291 K. The crystals were cryoprotected with 60 mM Citric Acid pH 4.0, 10% PEG3350 and 15% Glycerol and flash-frozen in liquid nitrogen.

### Data processing and structure determination

X-ray diffraction data were collected on Diamond beamline I04-1 for the hexagonal structure (*P*6_5_) and I24 for the triclinic structure (*P*1) (Diamond Light Source, Harwell, UK). Data were indexed and integrated with *iMOSFLM*^14^. Data reduction and scaling was accomplished using *SCALA*^15^ within the *CCP4* suite of programs^16^.

The first structure, which is in the triclinic form, was determined using *phenix.phaser*^17^. A chimeric model was applied as the probe for molecular replacement consisting of the heavy chain of the humanized RK35 antibody^18^ and the light chain of anti-ErbB2 Fab2C4^19^. The second structure in the hexagonal form was then solved based on a partially refined model obtained from the triclinic crystals. Refinements were carried out by *phenix.refine*^17^ iterated with real-space manual rebuild using *Coot*^20^. The first rounds of refinements were done in *phenix.refine* with rigid body and update water functions applied. Once a sharp decrease in *R*_*free*_ value had been observed, simulated annealing and translation–libration–screw (TLS) parameterization were included separately for systematic comparison. Refined models were manually rebuilt in *Coot* and iteratively refined by *phenix.refine*. The final data collection parameters and refinement statistics are shown in Table 1.

**Table 1:** Data collection and structure refinement statistics for A33 Fab in space groups P1 and P6_5_.

Crystal form	Triclinic	Hexagonal
Data collection		
Wavelength (Å)	0.9763	0.91188
Space group	*P*1	*P*6_5_
*a*, *b*, *c* (Å)	37.67, 69.73, 89.85	93.83, 93.83, 201.74
α, β, γ (°)	99.71, 101.43, 105.66	90, 90, 120
Resolution range (Å)	85.59–2.30 (2.38–2.30)	75.37–2.20 (2.28–2.20)
No. of reflections	106,776 (7689)	202,208 (26,490)
Unique reflections	35,070 (2710)	27,308 (2676)
Multiplicity	3.0 (2.8)	7.4 (6.8)
Completeness (%)	94.0 (72.9)	98.4 (90.3)
〈 *I*/σ(*I*)〉	11.0 (5.7)	12.1 (2.1)
*R*_merge_ (%)	5.6 (14.5)	9.6 (107.8)
*R*_meas_	0.068 (0.177)	0.103 (1.167)
Refinement		
Overall Wilson *B* (Å^2^)	31.8	36.7
R.m.s. deviations		
Bond lengths (Å)	0.012	0.011
Bond angles (°)	1.41	1.29
No. of atoms		
Protein non-hydrogen atoms	6608	3342
Water	267	166
B factors (Å^2^)	34.6	54.8
Protein	34.7	55.1
Water	32.1	48.1
*R*_work_/*R*_free_ (%)	19.4/27.1	20.5/25.6
Ramachandran		
Preferred, allowed, outliers (%)	94.4, 5.0, 0.6	93.1, 5.2, 1.7

Values for the outer shell are given in parentheses.

### Homology modelling

Some loops were too flexible to be resolved in the raw crystal structures and needed to be constructed. *RosettaCM*^21^ was used to fill those missing residues through a homology modelling protocol by applying the Fab A33 full-length amino acid sequences (SI) to the raw crystal structures of P1 and P6_5_. For each homology modelling, more than 20,000 structures were generated after the *RosettaCM Hybridize* step. The highest scoring structure with all five disulfide bonds intact was selected for the subsequent *RosettaCM Final relax* step. In the *Final relax* step, more than 20,000 structures were obtained and the highest scoring structure with all five disulfide bonds intact was selected. Jobs were submitted to the UCL Myriad High-Performance Computing Facility (Myriad@UCL) with Rosetta Version 2018.48.60516-mpi.

### Molecular dynamics

The Gromacs^22^ software was used to perform molecular dynamics, using the full-residue structures modelled based on *P*1 and *P*6_5_ crystals. The PDB2PQR (Dolinsky et al. 2004) webserver was used to determine the protonation states of ionisable residues at pH 4.5, pH 7 and pH 9 for *P*1 structure, and pH 7 and pH 4 for *P*6_5_ structure. The pH was maintained in simulations by the protonation state of the initial structure. Protonation states were not updated during the simulation. The simulation was carried out under *OPLS-AA/L* all-atom force field at 300 K and 1 bar. The protein molecule was placed within a cubic box with 1 nm distance between the protein and the box. The box was solvated with *SPC/E* waters, and Na^+^ and Cl^−^ ions to neutralise and provide ionic strength at 50 mM to the system. The system was then energy minimised and equilibrated around the solute protein, under an NVT (constant Number of particles, Volume, and Temperature) and an NPT (constant Number of particles, Pressure, and Temperature) ensemble. Production run was finally conducted for 100 ns. Six repeats were performed for each condition. Jobs were submitted to the UCL Myriad High-Performance Computing Facility (Myriad@UCL) using gromacs/2019.3/intel-2018.

### Elbow angle calculation

The elbow angle of the Fab is the angle between the pseudo-twofold axes between the variable and constant domains. It is calculated using the Pymol (Version 2.3.2) elbow_angle.py script, setting the V_L_ domain as residue 1–109 and V_H_ domain as residue 1–117. The elbow angles were calculated for the *P*1 and *P*6_5_ crystal structures, the free and binding states of the Certolizumab, as well as the trajectories from the molecular dynamics based on full-residue *P*1 and *P*6_5_ structures.

### SAXS curve fitting

The raw buffer-subtracted SAXS data of A33 Fab was derived from our previous work^11^. SAXS data collected in vitro at pH 4.5, 7 and 9 were used to compare the fitting from the five MD conditions as shown in Table 2. The PDB structure from the homology modelling step and MD trajectories from the molecular dynamics step were converted to dcd file format and submitted to the SASSIE webserver^23^ to obtain their corresponding SAXS curves, i.e. in-silico SAXS data. This was fitted against the in vitro SAXS data, using Chi-Square to indicate the fitness^23^. The top 10 best-fit structures were extracted into PDB format, aligned in Pymol and RMSD calculated by Gromacs.

**Table 2:** The MD trajectories used to fit to their corresponding SAXS data.

MD condition (6 repeats)	SAXS data (averaged from 3 repeats)
P65_pH4_50mM-IS_300K	4.5, 50 mM NaCl, 20 °C
P1_pH4.5_50mM-IS_300K	
P1_pH7_50mM-IS_300K	7, 50 mM NaCl, 20 °C
P65_pH7_50mM-IS_300K	
P1_pH9_50mM-IS_300K	9, 50 mM NaCl, 20 °C

## Results

### Overall description of A33 Fab structure

A33 Fab is a fully-humanised fragment consisting of a γ heavy chain and a κ light chain. Each chain contains a variable domain (V_L_ and V_H_) and a constant domain (C_L_ and C_H_1). The variable framework sequence was derived from the human antibody LAY and substituted with murine-derived CDRs while the constant domain fully originated from human immunoglobulin constant regions^1^. The elbow angles, defined as the intersection angle of the two pseudo-dyad axes (PDAs) between the variable domain and the constant domain, for the two structures solved in *P*1 and *P*6_5_ space groups were 156° and 145° respectively, which implied flexibility of the switch region^24^.

**Figure 1:**
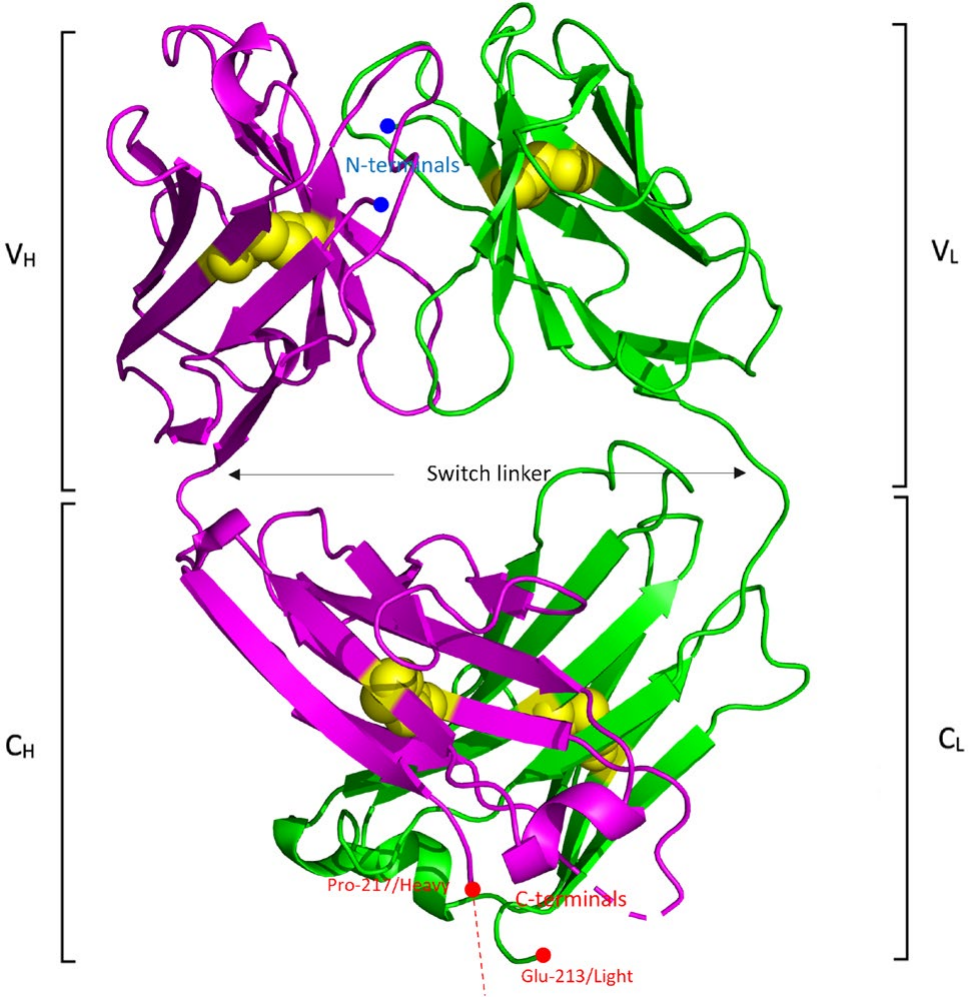
Overall A33 Fab structure. The heavy chain is coloured magenta while the light chain is coloured green. A33 Fab illustrates a canonical β-sandwich Ig fold within four domains (VL, VH, CL and CH1) and each domain contains a disulphide bridge between the inner and outer β-sheets (yellow sphere). Missing C-terminal region of the heavy chain (_heavy_K218 to _heavy_A228) is marked with a red dash. Image generated using PyMol v2.5: (https://pymol.org/2/).

A33 Fab illustrates a canonical β-sandwich Ig fold within four domains (V_L_, V_H_, C_L_ and C_H_1). Each domain has two layers of β-sheets, an inner- and an outer β-sheet. One canonical disulphide bridge was identified in each domain between the β-sheets layers (between _heavy_C22-_heavy_C96 in V_H_, _light_C23-_light_C88 in V_L_, _heavy_C144-_heavy_C200 in C_H_ and _light_C134-_light_C194 in C_L_), which contributes to the stability of the Fab (Fig. 1). An inter-chain disulphide bridge, _light_C214-_light_C220, was not resolved due to the missing loop in the hinge region of the heavy chain. Seven inter-chain hydrogen bonds have been identified between the heavy and light chains, which are _heavy_P102-_light_Y36, _heavy_F103-_light_T46, _heavy_W106-_light_T46, _heavy_Q39-_light_Q38, _heavy_Q39-_light_Q38, _heavy_P170-_light_S162 and _heavy_P126-_light_S121. In addition to hydrophobic interactions, there are five inter-chain hydrogen bonds in variable regions while only two in the constant domains, illustrating tighter contacts within the variable regions.

### Comparison of A33 Fab structures in different space groups

**Figure 2:**
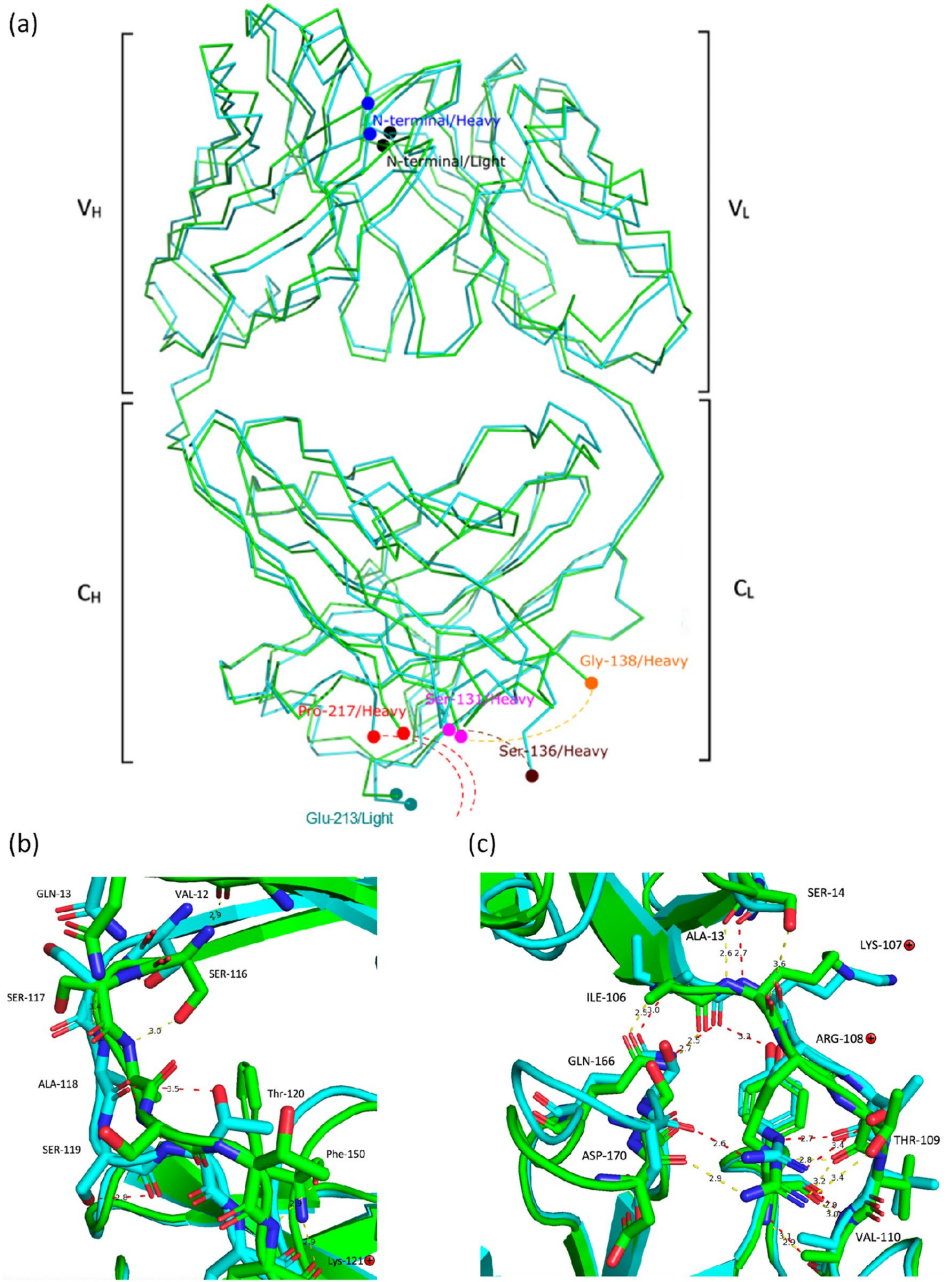
(**a**) Superposed C^α^ trace of A33 Fab structures in space groups P1 (green) and P6_5_ (cyan). The missing regions (_heavy_S132 to _heavy_T135 and _heavy_K218 to _heavy_A228) are marked with dash lines. The two structures share high similarity but display differences in the switch region. (**b**) Superposed heavy chain switch region. The triclinic model (P1) is coloured in green while the hexagonal model (P6_5_) is coloured in cyan. Conformational differences can be viewed from _heavy_S117 to _heavy_S119 while light chain switch peptides are fixed by a set of hydrogen bonds. The protonated residues have been marked with cross markers. (**c**) Superposed light chain switch region. Images generated using PyMol v2.5: (https://pymol.org/2/).

The triclinic crystals (*P1*) diffracted to 2.5 Å resolution with two highly similar copies (RMSD = 0.339 Å) of A33 Fab per asymmetric unit (AU), while the hexagonal crystals (*P6*_*5*_) had only one copy per AU and diffracted to 2.2 Å. The overall structures in both crystal forms were similar but displayed slight differences (Fig. 2a). The main difference between the two structures was the elbow angle. Different compact patterns affected the intersection angle between the variable region and the constant region. The elbow angles shifted from 156° in the triclinic form to 145° in the hexagonal form, which implied flexibility of the switch region. However, the variable regions and the constant regions shared great similarity. The RMSD calculated between C^α^-atoms of matched residues at 3D superposition of the two structures was 1.33 Å. In contrast, the RMSD decreased to only 0.5 Å and 0.76 Å for separately superposed variable- and constant regions, respectively.

Closer insight into the linkers between the variable and the constant regions revealed conformational differences between the two models in the heavy-chain switch regions. The side chains of _heavy_S117 and _heavy_S119 had different orientations and formed different hydrogen bonds with adjacent residues. Also, two unique hydrogen bonds were formed between _heavy_A118 and adjacent residues in the hexagonal model. In contrast, the linker in the light chain was fixed by a set of hydrogen bonds, which granted it less flexibility (Fig. 2b,c). The PDB2PQR web server was used to calculate the pKa for ionisable linker residues. However, there were no discernible differences that would alter their protonation states between the *P*1 and *P*6_5_ models in the linker region, indicating that protonation has a limited impact on the conformational differences observed. As such it remains unclear whether the structural differences in the switch linker region were as a result of the pH or the differences in crystal packing.

Two loops were missing in both structural models, which indicated high flexibility in this region. In the C_H_ domain the missing loop was composed of four residues (SKST, _heavy_S132 to _heavy_T135) in the hexagonal model and six residues (SKSTSG, _heavy_S132 to _heavy_G137) in the triclinic model. The hydrophilicity of the missing loop could facilitate interactions with solvents and thus, contribute to the flexibility of the loop. The other missing loop was located at the C-terminus of the heavy chain, which was also the hinge region of A33 Fab. The missing loop in this region contained 11 residues (KSCDKTHTSAA, _heavy_K218 to _heavy_A228).

### Humanised complementarity determining regions (CDRs)

A33 Fab contains a highly humanised variable region composed of human variable framework regions (FWRs) from the LAY antibody and murine CDRs to minimise the formation of human anti-chimeric antibodies (HACAs)^1^.

**Figure 3:**
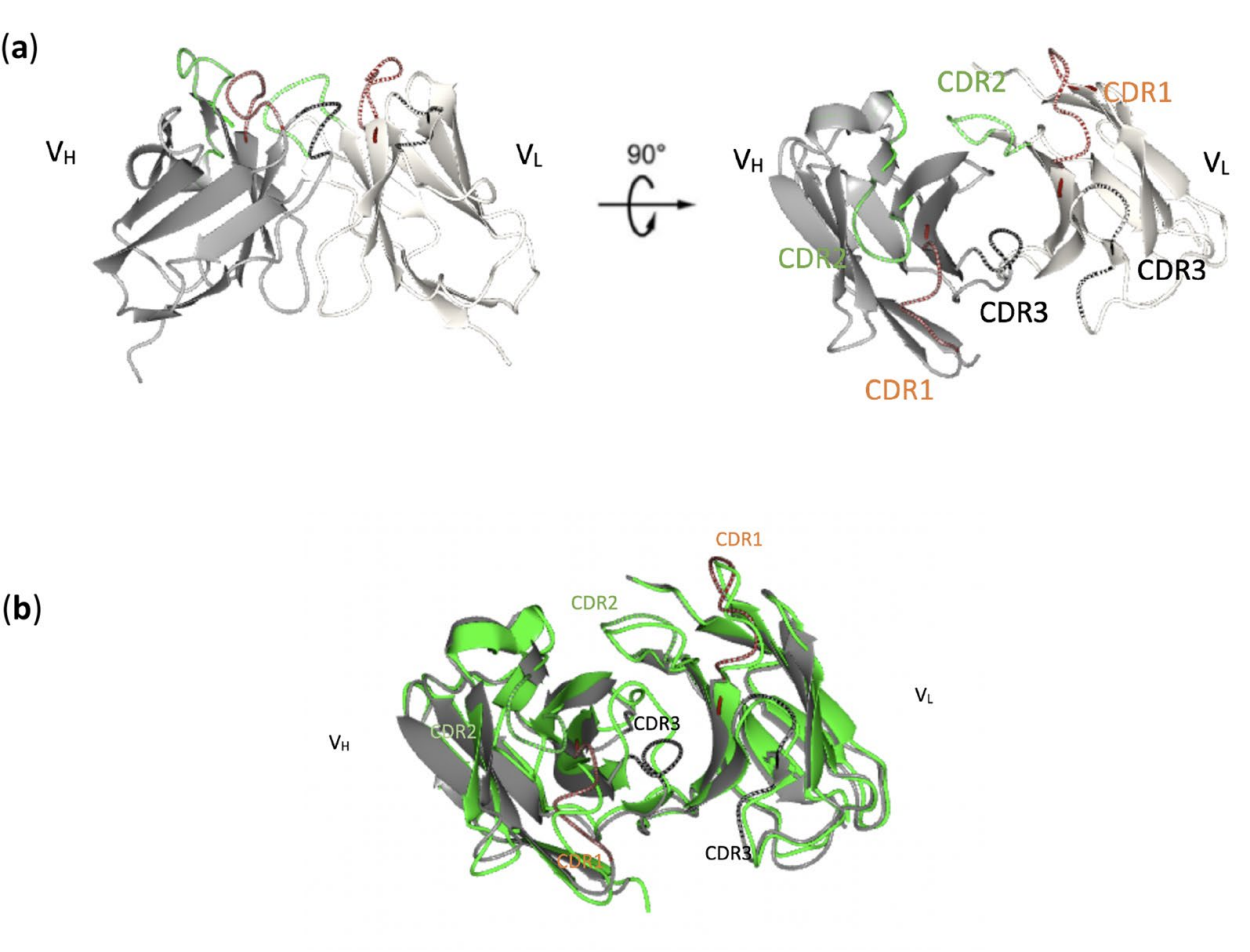
CDRs of A33 Fab. (**a**) CDR1 (brown), CDR2 (green) and CDR3 (black) originating from monoclonal murine A33 Fab are responsible for antigen binding. (**b**) Superposition of A33 Fab (grey) with Certolizumab (green). Images generated using PyMol v2.5: (https://pymol.org/2/).

Here we aligned the A33 Fab for comparison with the peer therapeutic antibody Certolizumab which targets a different antigen^25^. Certolizumab and A33 Fab have been developed from the same humanization scaffold with high sequence similarity (Supplementary, Fig. S1). The constant regions of A33 Fab and Certolizumab share high similarity. The RMSDs for C_H_ and C_L_ regions between A33 Fab and Certolizumab are 0.65 and 0.49 Å, respectively. By aligning the paratope regions, major differences can be found in CDR2 in V_L_ domains, CDR3 and CDR2 in V_H_ domains. Despite the large variation between CDRs, the FWRs remain highly similar (Fig. 3a,b), which implies the rigidity of the FWRs.

### Molecular dynamics (MD) probed distinct Rg and APR/SASA at different pH

Two crystal structures for A33 Fab were obtained at different pH, namely the *P*1 form at pH 9 and the *P*6_5_ form at pH 4. However, it is unknown as to how closely these structures match the solution structures of Fab at various pH derived from SAXS and/or MD simulations. Potentially the two crystal structures would also provide improved starting points for MD simulations at a range of pH. To investigate this, the two crystal structures were used to investigate their respective impact on MD simulations at different pH, and the range of solution structure conformations explored by each simulation. These were then also compared with SAXS data available from a previous study at each pH^11^. This would shed light on the importance of the choice of starting structure on subsequent MD analyses at various pH, and also compare the impact of pH on the structures obtained through all three approaches.

**Figure 4:**
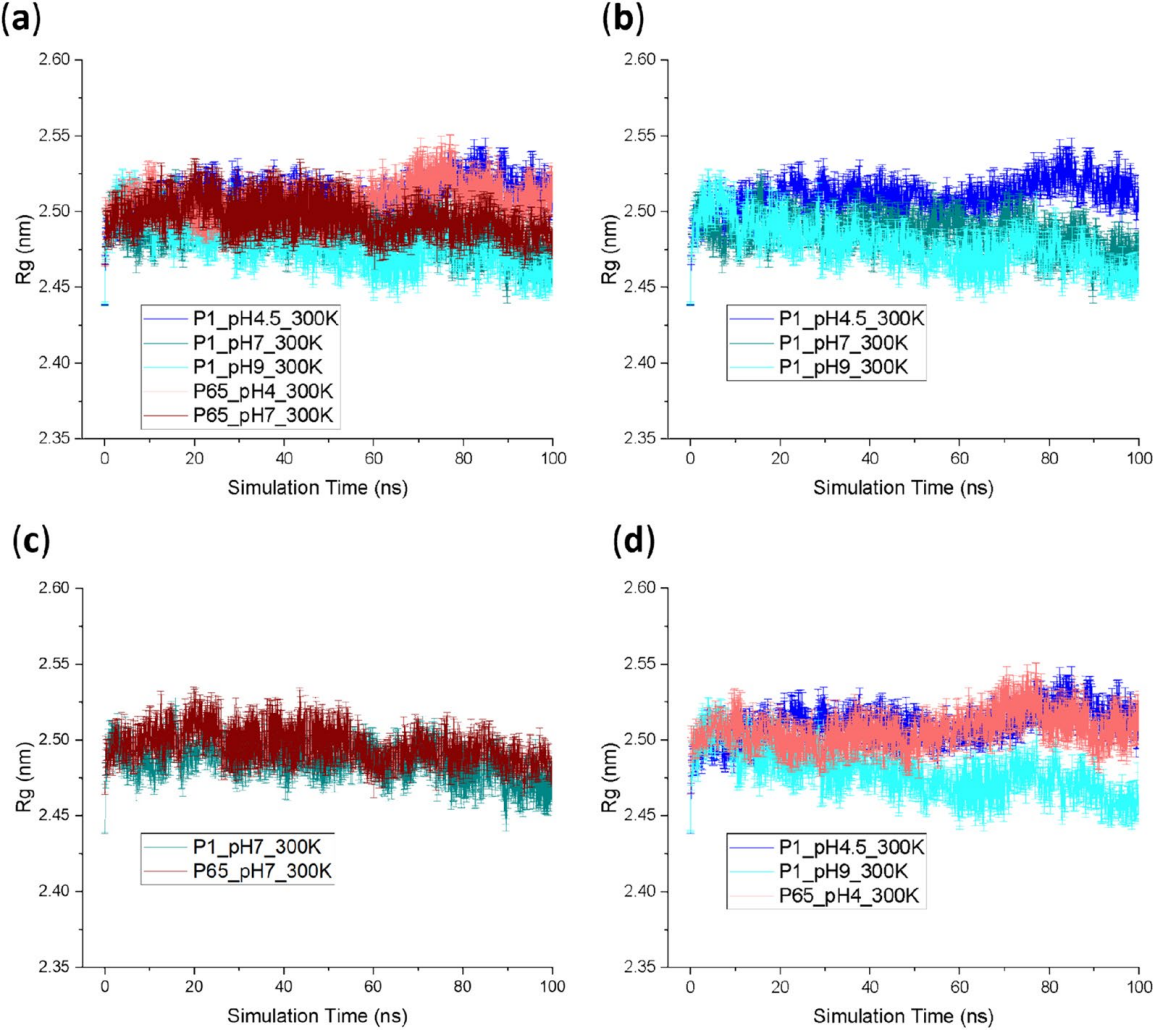
Radius of gyration (Rg) of Fab starting from different crystal structures at different pH during the simulation. The Rg of all the five conditions were superimposed in (**a**) while fewer conditions were plotted separately in (**b**–**d**) for the ease of comparison. Six repeats were performed for each unique condition, and the average values were reported with standard error of the mean (SEM) as the error bars.

The impact of starting structure and pH on the total protonation states of the protein are reported in Supplementary Table S1, and show that pH 7 leads to lower total charge (+ 7) as expected than for pH 4 (+ 17 to + 26). *P*6_5__pH4 (+ 26) carried a greater charge than P1_pH4.5 (+ 17), indicating some structural differences that affected surface ionisable groups and/or salt bridges. As shown in Fig. 4, all five conditions started with similar Rg values (2.44–2.46 nm) but diverged towards the end of simulations. At 100 ns, the two low pH conditions (*P*1_pH4.5, *P*6_5__pH4) both achieved the highest Rg at more than 2.51 nm, followed by the two neutral pH conditions (*P*1_pH7, P6_5__pH7) at 2.47–2.48 nm, and with the high pH condition (*P*1_pH9) having the lowest Rg at 2.46 nm. This corresponded well to our previous experimental SAXS data^11^, where the Fab shared comparable Rg at pH 7 and pH 9, but had much higher Rg at pH 4.5. The similar ranking of end-point Rg at various pH was clearly the result of their respective protonation states in the MD simulation, and was unaffected by any initial crystallographic bias from the two crystal structures of *P*1 and *P*6_5_.

**Figure 5:**
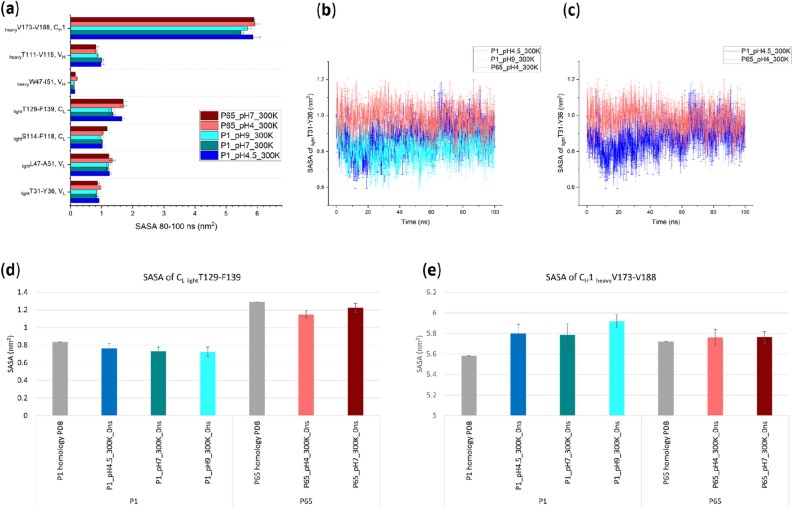
(**a**) The average Solvent Accessible Surface Area (SASA) of representative aggregation-prone regions (APR) in the final 20 ns of simulations, in response to various pH from different crystal structures; (**b**, **c**) APR SASA of residue _light_T31-Y36 during the simulation; (**d, e**) The SASA of residue _light_T129-F139 and residue _heavy_V173-V188 in the *P*1, *P*6_5_ homology models and the MD conditions at time 0;. Error bars are SEM (n = 6 repeats) and are equal for positive and negative values.

The increased Rg at low pH suggested a possible conformational instability of the A33 Fab, that exposed more aggregation-prone regions (APR) to the solvent and resulted in tenfold faster in vitro aggregation at pH 4 compared to at pH 9^5^. Therefore, we evaluated all seven APRs of A33 Fab predicted previously^5^ and their different responses to the various pH stresses, based on the current simulations starting from the two different crystal structures (Fig. 5a). This showed that at the end of the simulation (80–100 ns), *P*1_pH9 had the lowest Solvent Accessible Surface Area (SASA) in nearly all the APRs, while *P*1_pH4.5 and/or *P*6_5__pH4 saw increased SASA at four APRs located at residues _heavy_V173-V188 (C_H_1), _light_T129-F139 (C_L_), _light_L47-A51 (V_L_) and _light_T31-Y36 (V_L_). This implies an increased Fab light chain volatility under low-pH stresses.

**Figure 6:**
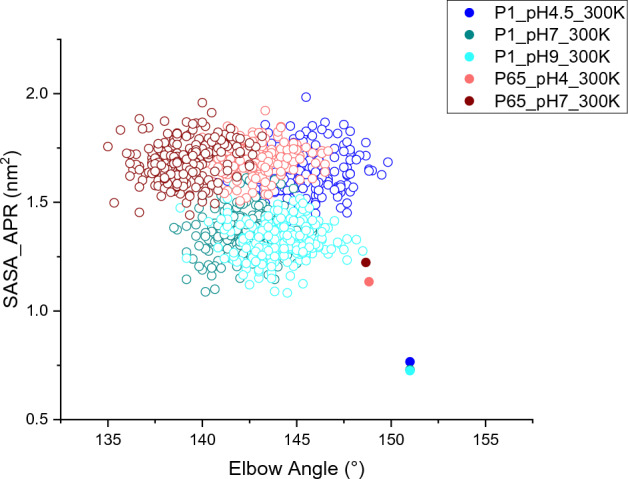
The SASA of C_L_ residues _light_T129-F139 and corresponding elbow angles during the 80–100 ns (hollow circle) and 0 ns (filled circle). At 0 ns, the *P*1_pH7_300K data point (green filled) is covered by the *P*1_pH9_300K one (cyan filled).

It is noted that for C_H_1 residues _heavy_V173-V188 and C_L_ residues _light_T129-F139, the end-point SASA for P6_5_ simulations were clearly higher. For C_L_ residues _light_T129-F139, this was likely due to the fact that the starting SASA (0 ns) of P6_5_ C_L_ residues _light_T129-F139 were already 25% higher than those of *P*1 (Fig. 5d). By contrast, C_H_1 residues _heavy_V173-V188 had comparable starting SASA between *P*1 and *P*6_5_ (Fig. 5e). Therefore, for C_L_ residues _light_T129-F139 there appeared to be a persistent difference between the *P*6_5_ and *P*1 structures within this region. The distribution of SASA and elbow angles for each simulation in the final 20 ns, compared to their starting structures (Fig. 6) reveals that both *P*1 and *P*6_5_ simulations moved towards decreased elbow angles, and increased SASA for C_L_ residues _light_T129-F139. However, *P*1 SASA and elbow angles remained offset from those of *P*6_5_, by a similar amount found between their starting structures. Indeed the *P*1 simulations appeared to have only just converged on and then passed the *P*6_5_ starting position in the final 20 ns of the 100 ns simulations. Thus, the *P*6_5_ starting structure was already a better representation of the solution structure at pH 4 for residue _light_T129-F139, than was *P*1. However, it is expected that for the C_L_ residues _light_T129-F139, the *P*1 trajectories would also eventually converge with the *P*6_5_ trajectories at each respective pH given longer simulations. Only for this specific APR did the solvent accessibility depend on the starting structure used.

In addition, the evolution of APR residues _light_T31-Y36 can be more clearly visualised in Fig. 5b,c. This shows that the end points converged for *P*1_pH4.5 and *P*6_5__pH4 in this region (Fig. 5c), while *P*1_pH9 retained its divergence from *P*6_5__pH4 (Fig. 5b), indicating the tendency towards increased exposure of this APR at low pH. Furthermore, the initial conformations of *P*1 and *P*6_5_ were already good representatives of their respective crystallographic pH for this APR, as the SASA from MD simulations at the same pH did not change significantly.

### RMSD, RMSF and elbow angle did not reveal crystal structure and pH dependency

**Figure 7:**
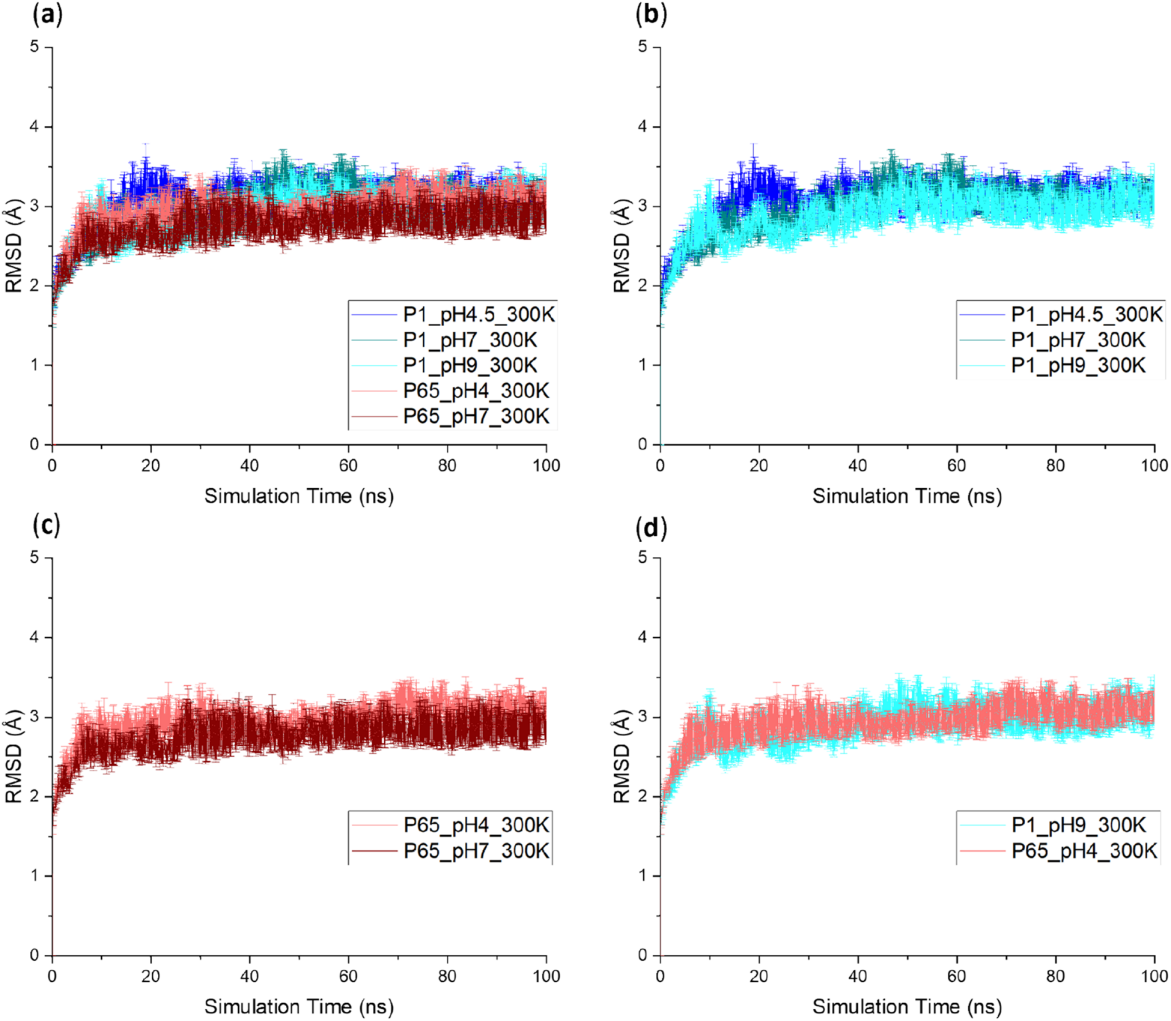
Root mean square deviation (RMSD) of Fab starting from different crystal structures at different pH during the simulation. The RMSD of all the 5 conditions were superimposed in (**a**) while fewer conditions were plotted separately in (**b**–**d**) for the ease of comparison. Six repeats were performed for each unique condition, and the average values were reported with standard error of the mean (SEM) as the error bars.

**Figure 8:**
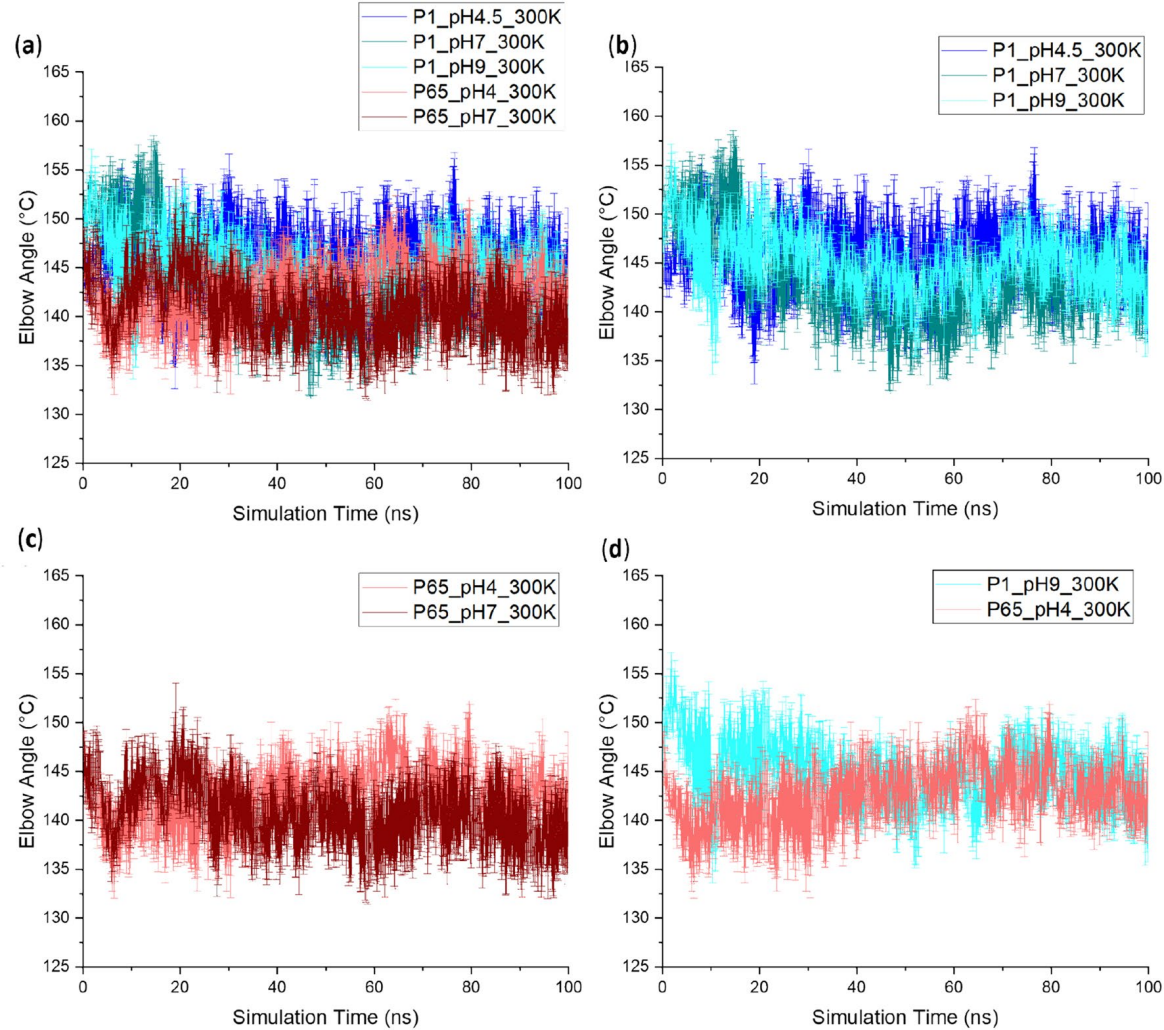
Elbow angle of Fab starting from different crystal structures at different pH during the simulation. The elbow angle of all the 5 conditions were superimposed in (**a**) while fewer conditions were plotted separately in (**b**–**d**) for the ease of comparison. Six repeats were performed for each unique condition, and the average values were reported with standard error of the mean (SEM) as the error bars.

RMSD (Fig. 7), elbow angle (Fig. 8) and RMSF (Supplementary Fig. S2) did not reveal significant differences at the end of the MD performed at the different pH. Initial changes seen in the first 3 ns are typical of the system continuing to equilibrate into the solution conditions as seen previously^5^ and can be ignored. RMSD was relatively insensitive to either pH or starting structure conditions compared to Rg or the SASA of certain APRs. Residue-level RMSF was analysed at each 10 ns interval for the four Fab domains independently, and was largely unaffected by either pH or starting structure. Exceptions, shown for the final 10 ns interval in Supplementary Fig. S2, were at residues 349–350 and 404–410 in the C_H_1 domain for which RMSF values were higher for *P*6_5_ simulations than for *P*1 simulations (regardless of pH). These two solvent exposed regions interact in the structure close to the C-terminal hinge end of the molecule and bury a significant portion of the APR _light_T129-F139. This increased flexibility at residues 349–350 and 404–410 within the *P*6_5_ simulations therefore accounts for the increased SASA for APR _light_T129-F139 also observed in these simulations.

Also, the low pH appeared to increase RMSF within a small region at residues 199–204 in the C_L_ domain for both the *P*1 and *P*6_5_ simulations. This demonstrates again that the starting structure did not significantly influence the simulations, giving consistent effects due to changes in the pH.

For the elbow angle, while the starting angles varied in the range 147°–151°, simulations from all conditions converged the elbow angles to around 139°–145° in the last 20 ns. *P*1_pH9 and *P*6_5__pH4 with initial elbow angles of 151° and 148.8° respectively, were simulated at their respective crystallisation pH conditions as shown in Fig. 8d. In the *P*1_pH9 structure, the angle dropped to 141° at 10 ns, before rising back to 146° at 20 ns, then settling out at between 140° and 145°. By comparison, the *P*6_5__pH4 structure witnessed a much smaller decrease in elbow angle, with a short-lived excursion to 148.8° at 62.5 ns, but mostly remaining within the 138°–145° range. This implied that the *P*6_5_ crystal structure resolved at pH 4 had an elbow angle more similar to those in solution at any pH, while the elbow angle for *P*1 was possibly due to a conformation that is only rarely populated in solution, even at pH 9, and yet became stabilised by crystallisation at pH 9. It also suggests that for the elbow angle at least, the starting structure is much less critical than the influence of pH configuration on the end-points of MD simulations, and that longer simulations would likely remove any influence of starting structure. This does however, highlight that where the aim is to use MD simulation data taken from throughout the trajectory to evaluate protein structure and dynamics, then the starting structure does influence the outcome initially, and so care should be taken to run longer simulations, or to compare multiple starting points.

### SAXS curve fitting revealed representative MD conformations at pH 7

**Figure 9:**
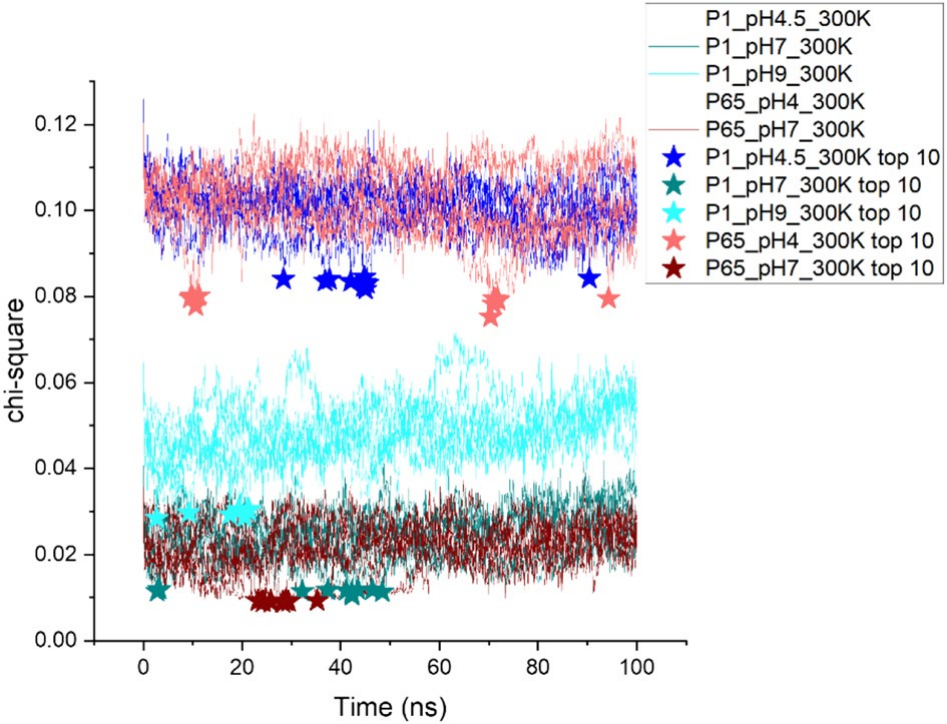
Goodness of fit (Chi-square) between experimental SAXS curves and all frames during Fab MD simulations with different pH and starting crystal structures. Six repeats of each MD condition were plotted in the same colour as previous figures. The top 10 best-fit structures are highlighted as stars to denote their occurrence in simulation time.

The fitness of the MD trajectories to the in vitro experimental SAXS data is shown in Fig. 9. The two pH 7 conditions gave the best fits of around 0.02 chi-square, followed by the pH 9 condition with 0.05 chi-square, while the pH 4/4.5 simulations gave the lowest fitness of 0.10 chi-square. It is clear that the ranking in the SAXS fitting heavily depended on the pH, regardless of the initial crystal structure used, and that the simulations at pH 4.5 still had a long way to go until they accessed the structures observed by SAXS experiments at low pH. Clearly the low-pH solution structures were also very different to the crystal structure *P*6_5_ obtained at pH 4, as well as to *P*1 obtained at pH 9.

The top 10 best-fits for each condition were highlighted as stars in Fig. 9. This shows that for the good-fit trajectories (pH 7 and pH 9), the best fits all occurred during the first half of the MD simulations. However, most of the best-fits arose in a single repeat for each condition, and occurred as outliers in the data within a 20 ns timeframe. In other words they were obtained from relatively rare events in the MD simulations, accessed as rapid fluctuations from a structural state that itself only existed for specific transient periods of approximately 20 ns. Again this type of event clustering was similar for both *P*1 and *P*65-based simulations, and so for application to SAXS fitting, the specific starting structure used in MD simulations was not a critical factor.

Even though *P*1 and *P*6_5_ were crystallised at pH 9 and pH 4, respectively, neither one of them outperformed the other by revealing conformations during MD simulations with better fitting to SAXS data. At any given pH, the simulations from the two different starting structures had essentially converged within the 100 ns timeframe sampled, and so were unlikely to lead to any differences if sampled for longer or with more advanced sampling methods.

For each MD condition, the chi-square fits were also plotted against Rg for all the trajectory frames (Supplementary Fig. S3a–e), to determine whether a minimum was reached which would indicate that sufficient sampling of states had been achieved to correctly model the SAXS data. Only the two pH 7 simulations reached such a minimum in the Chi-square vs Rg plot, with the simulation from *P*6_5_ at pH7 reaching the best fit overall. As no minimum was observed for pH 4/4.5 or 9, then either much longer simulations would be needed or otherwise more advanced sampling methods to fully resolve the best fit structures from the in vitro SAXS data at those pH. Indeed, better fits to the SAXS data were obtained in our previous work by increasing the temperature to 380 K, which enabled the simulations to explore more extensive structural changes in the MD^11^.

## Discussion

Over the past decades, significant efforts have been undertaken into the development of therapeutic antibodies against CRC targeting human antigen A33. Various A33 Fab variants have been developed and multiple clinical trials have been carried out^3,26^. However, various A33 Fab variants have failed in clinical trials due to the normal gut localization and intrinsic stability issues of the variants^11,26^. To our knowledge, the structure of A33 Fab or its variants has never been reported. Here we determined the structure of the highly characterised A33 Fab mutant, H/C226S, in two crystal forms.

The structural analysis of A33 Fab in two different crystal forms disclosed the intrinsic rigidity of four separate regions (V_L_, C_L_, C_H1_, C_L_) and the flexibility of the switch region. The different compact patterns and the interactions with solvents altered the elbow angles between the two pseudo-dyad axes from 156° (triclinic form) to 145° (hexagonal form), which led to a noticeable RMSD between the structures obtained from the two crystal forms. In contrast, the canonical β-sandwich Ig folds contributed to the rigidity of both variable and constant regions, which led to the high similarity of separate domains in different crystal forms.

We hypothesised that the major differences between the A33 Fab and Certolizumab were located at the variable region. Certolizumab was developed from the same humanization scaffold as A33 Fab, possessing a similar variable region framework and the same constant region protein sequence. To our surprise, the variable regions of these two Fabs shared higher similarities than expected (RMSD 0.91 Å). Only the V_H_ CDR3 and V_H_ CDR2 of both Fabs varied significantly, while the rest of the CDRs had similar conformations even with different protein sequences, which suggested the key roles of V_H_ CDR3 and V_H_ CDR2 in the identification between human A33 and TNFα. The FWRs were also similar between the two structures despite the sequence differences, which implied that the FWRs offer good support to the CDRs while being neutral to scaffolding for antibody structural integrity. The other main difference was the elbow angle. According to the previously published Certolizumab structure, the elbow angle of Certolizumab changed 9° during antigen binding, from 138° (free) to 129° (binding)^25^. In contrast, the elbow angles of *P*1 and *P*6_5_ structures of A33 Fab were 156° and 145° respectively. Certolizumab and Fab A33 possess the same protein sequence in switch and constant regions while the different elbow angles implied flexibility in the switch region. It has been reported that the flexibility of elbow angle is vital for the binding affinity of Fabs to antigens by optimizing antibody conformational dynamics and adaptation to antigen structure^27,28^. In conclusion, the scaffold of the A33 Fab is optimal to support CDRs targeting different antigens and has the potency to be applied to other therapeutic candidates from an engineering perspective. However, the pH-induced changes in elbow angle for A33 Fab do not appear to relate to the elbow-angle changes induced by ligand binding in Certolizumab.

The two structures could not resolve two flexible regions, including residues _heavy_S132 to _heavy_T135 and _heavy_K218 to _heavy_A228. Thus, homology modelling was conducted to predict the missing residues, followed by molecular dynamics to study their impact on the conformational changes at different pH.

Overall, the structural differences between *P*6_5_ and *P*1 were small in terms of their impact on MD simulations, as reflected by their comparable SAXS fitting and overall RMSD regardless of pH (Supplementary Fig. S3f) when comparing the top 10 best-fits. In addition, most of the APR solvent exposure was not impacted by the starting structure during the simulations. The only observed impacts of the starting structures were:

- The solvent exposure of APR _light_T129-F139, which remained different between *P*1 and *P*6_5_ simulations after 100 ns. *P*1 simulations lagged behind the *P*6_5_ simulations but after 100 ns they had at least passed through the solvent exposure (and elbow angles) found in the *P*6_5_ starting structure.
- *P*6_5_ achieved slightly better fits than *P*1 at pH 7 and pH 4.5, as it was already closer in structure to the solution forms.

Finally, it was interesting that the crystal structures *P*1 and *P*6_5_ obtained at pH 9 and pH 4, respectively, were not biased towards the solution structures indicated by SAXS under the equivalent conditions. However, both of the starting structures were already reasonable approximations of the pH 7 SAXS structure (Chi-square around 0.04), with *P*6_5_ marginally better than *P*1. This is an indication that crystallisation stabilised against the partial denaturations found in acidic or alkaline solutions, and instead selected out structures closer to those found at pH 7, despite the crystallisation at pH 9 or pH 4.

Successful development of protein therapeutics depends critically on achieving stability under a range of conditions. Starting from the crystal structure of A33 Fab in the *P*1 space group, we have previously compared the impacts of low pH and high temperature stresses on the structure of a humanized antibody fragment (Fab) A33, using atomistic molecular dynamics simulations^5^. An extensive dataset now exists for the stability and aggregation behaviour of A33 Fab variants and formulations, from which a deeper understanding of these mechanisms can be potentially gained by exploring MD simulations in more detail. The crystal structures of human A33 Fab, and the present finding that the specific starting structure chosen actually has little influence on the MD simulations at a range of pH, especially beyond the first 80 ns, will now provide a solid support for this testing ground.

### Supplementary Information

Supplementary Information.

## Data Availability

Coordinates for the A33 Fab models 7NC0 and 7NFA have been deposited and released in the PDB. Other data reported here are available on request to the corresponding author.
