# Supplementary material for: Crystal structures and molecular dynamics simulations of a humanised antibody fragment at acidic to basic pH
Source: Sci Rep. 2023 Sep 28;13:16281. doi: 10.1038/s41598-023-42698-7 (PMC10539359; doi:10.1038/s41598-023-42698-7)
Supplement: Supplementary file 1 — Supplementary Information. [file 41598_2023_42698_MOESM1_ESM.docx]

**Supplementary Information:**

**Crystal structures and molecular dynamics simulations of a humanised antibody fragment at acidic to basic pH**

Jiazhi Tang^1^, Cheng Zhang^2^, Nuria Codina Castillo^2^, Christophe Lalaurie^2^, Xin Gao^3^, Paul Dalby^2^*, Frank Kozielski^1^*

^1^UCL School of Pharmacy, 29-39 Brunswick Square, London, WC1N 1AX, UK,

^2^Department of Biochemical Engineering, UCL, Bernard Katz Building, Gower Street, London, WC1E 6BT, UK

^3^ Department of Structural and Molecular Biology, Division of Biosciences, UCL, London, WC1E 6BT, UK

Correspondence*: [f.kozielski@ucl.ac.uk](mailto:f.kozielski@ucl.ac.uk); [p.dalby@ucl.ac.uk](mailto:p.dalby@ucl.ac.uk)


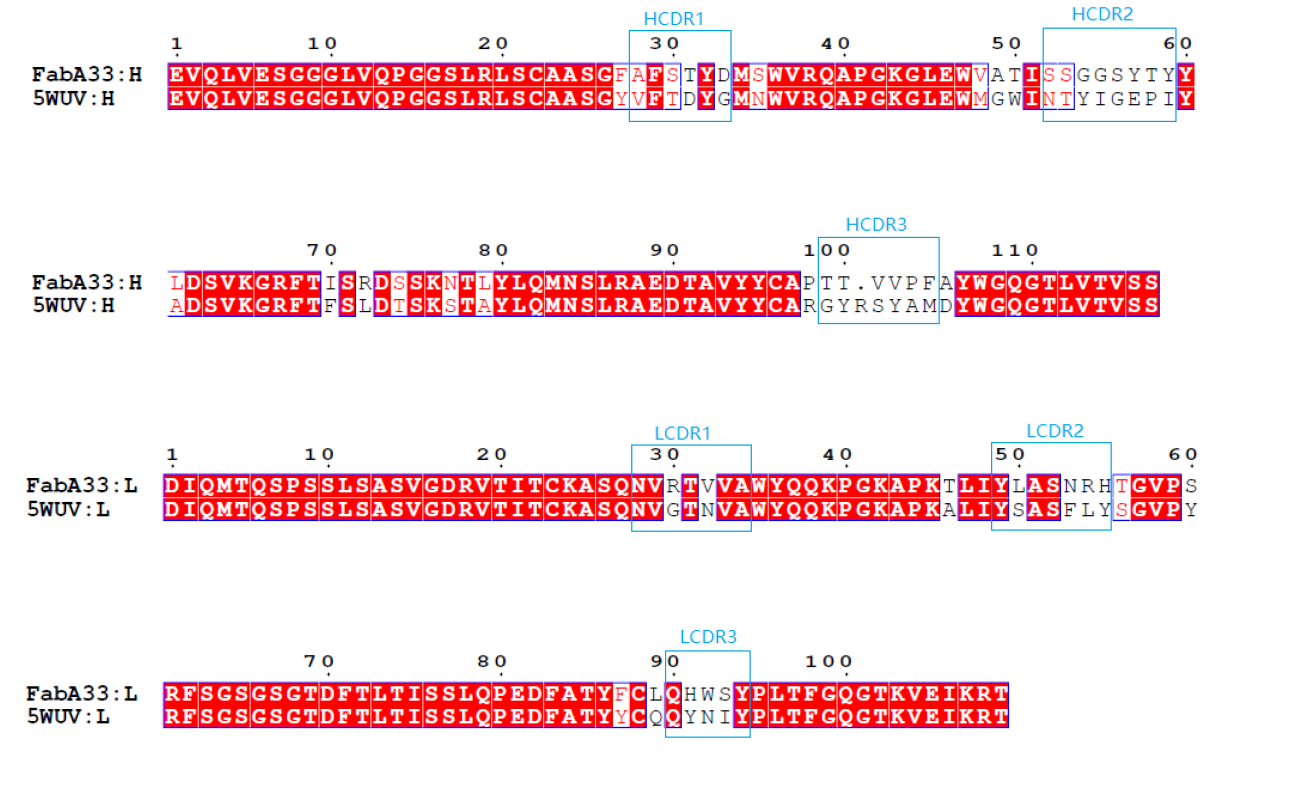


**Figure S1.** Alignments of variable domains between A33 Fab and Certolizumab.

**Table S1: Protonation states of starting structures used in MD simulations**

| **Space Group and MD pH** | **Total charge** |
| --- | --- |
| P1_pH4.5 | 17 |
| P1_pH7 | 7 |
| P1_pH9 | 7 |
| P65_pH7 | 8 |
| P65_pH4 | 26 |

Protonation states were calculated using PDB2PQR

**Figure S2.** The root-mean-square fluctuation (RMSF) for each residue in the **A)** V_L_, **B)** C_L_, **C)** V_H_, and **D)** C_H_1 domains during the final 10 ns (90-100 ns interval) of the simulations. The displayed values are the averages derived from six independent repeats, with error bars indicating the standard error of the mean (SEM).


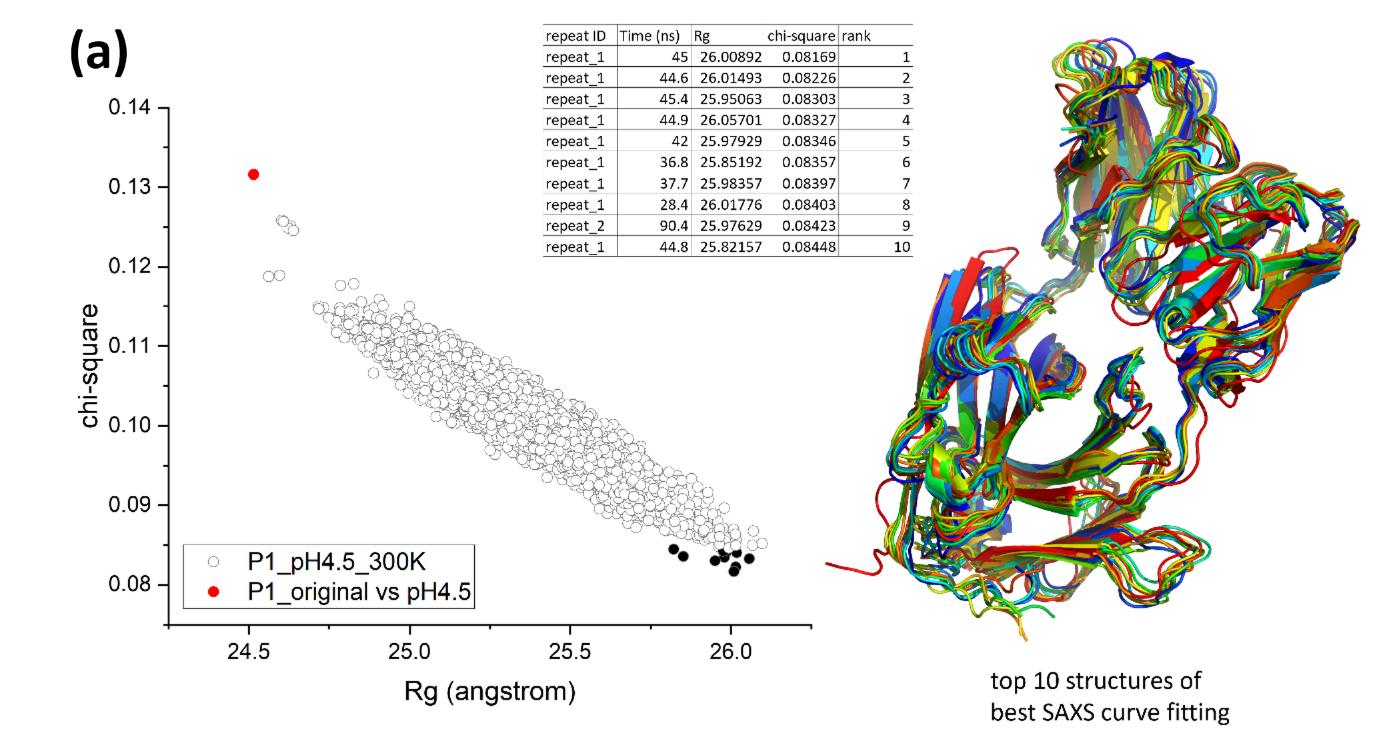


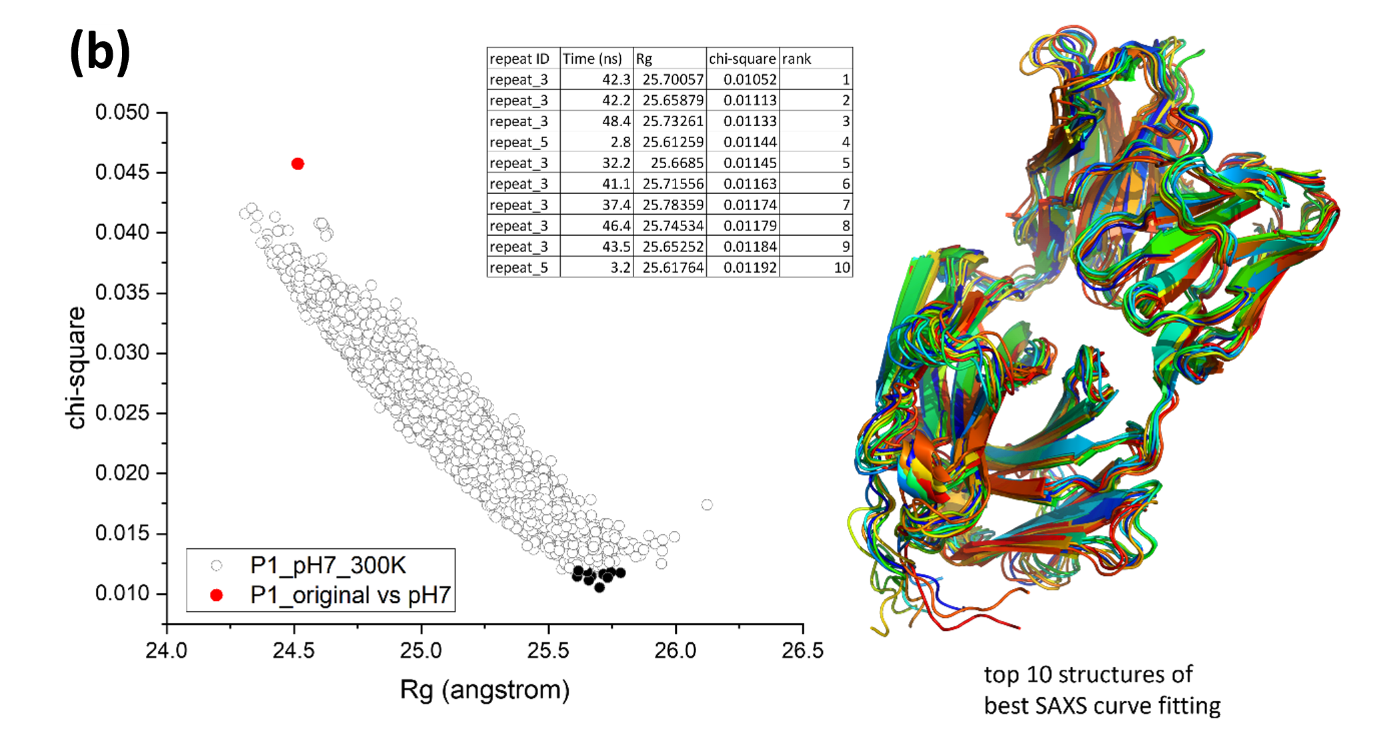


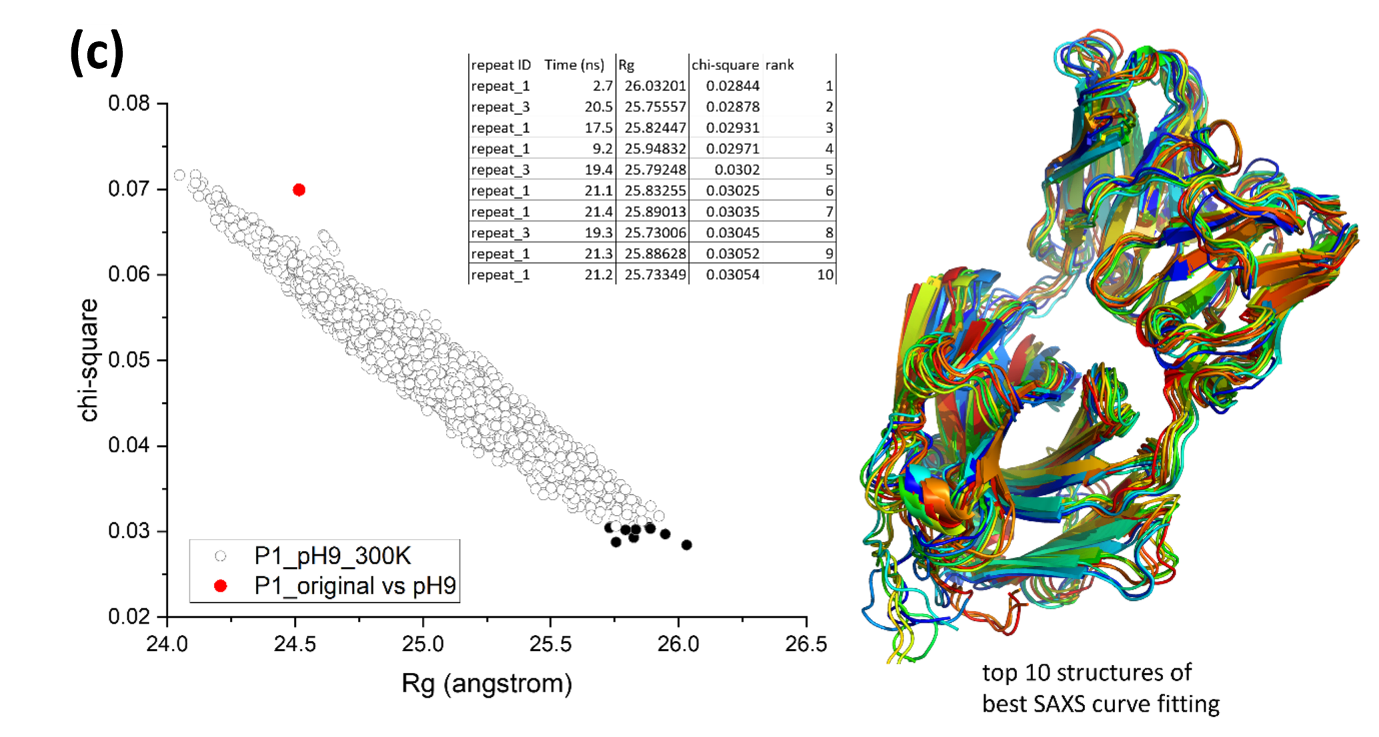


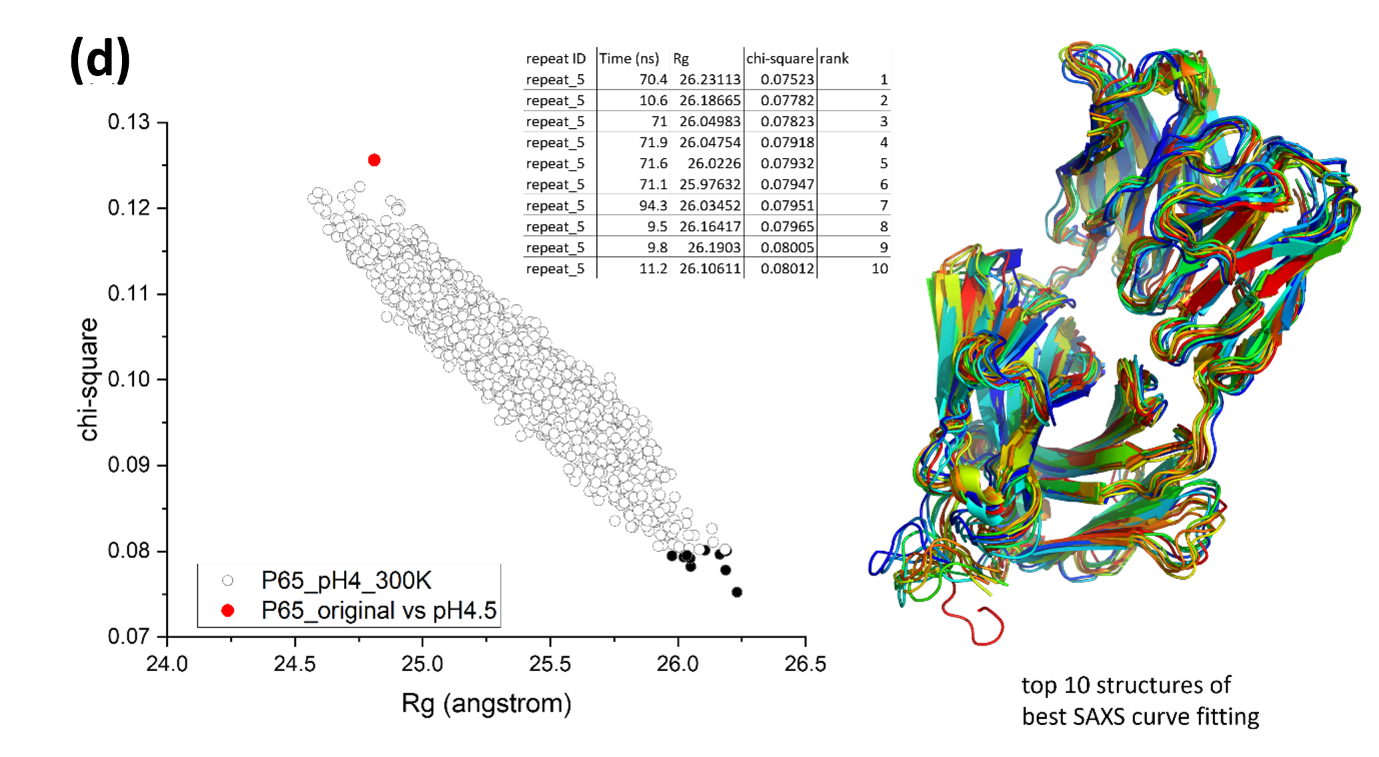


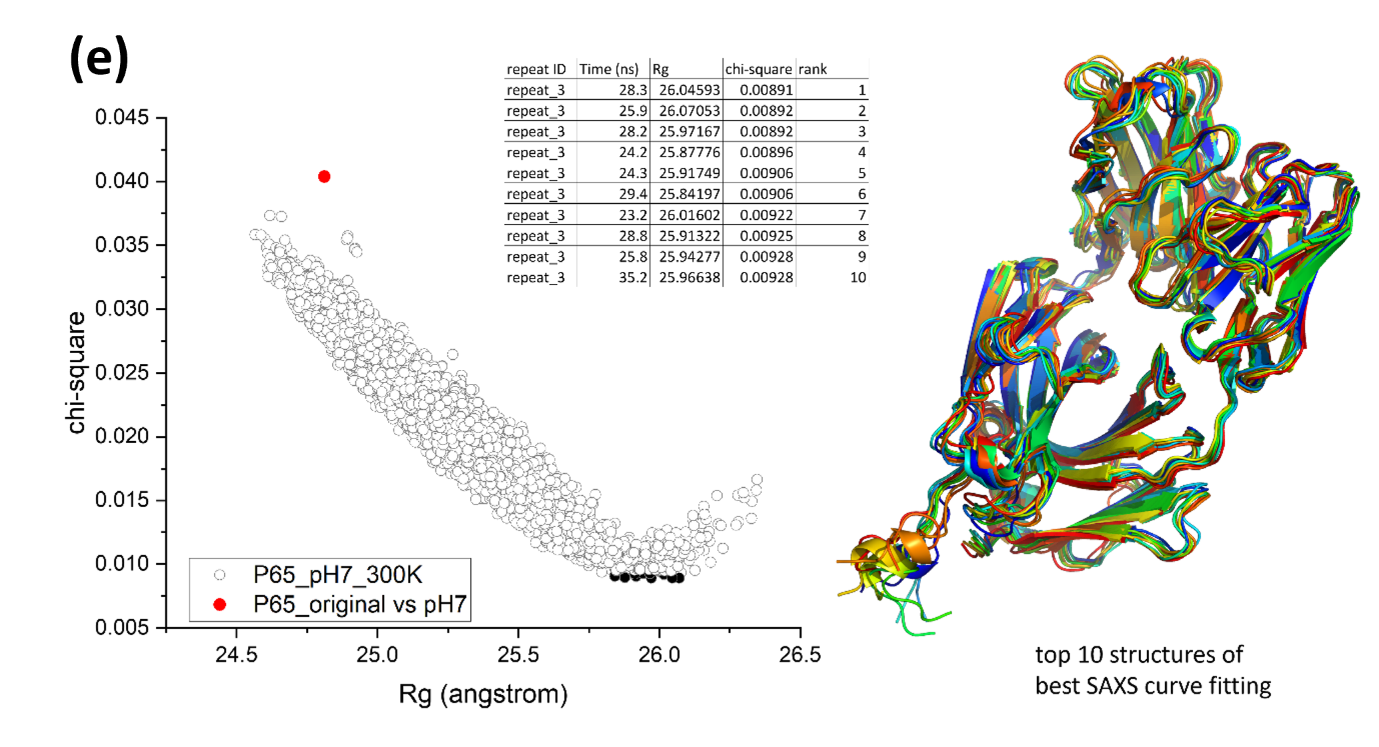


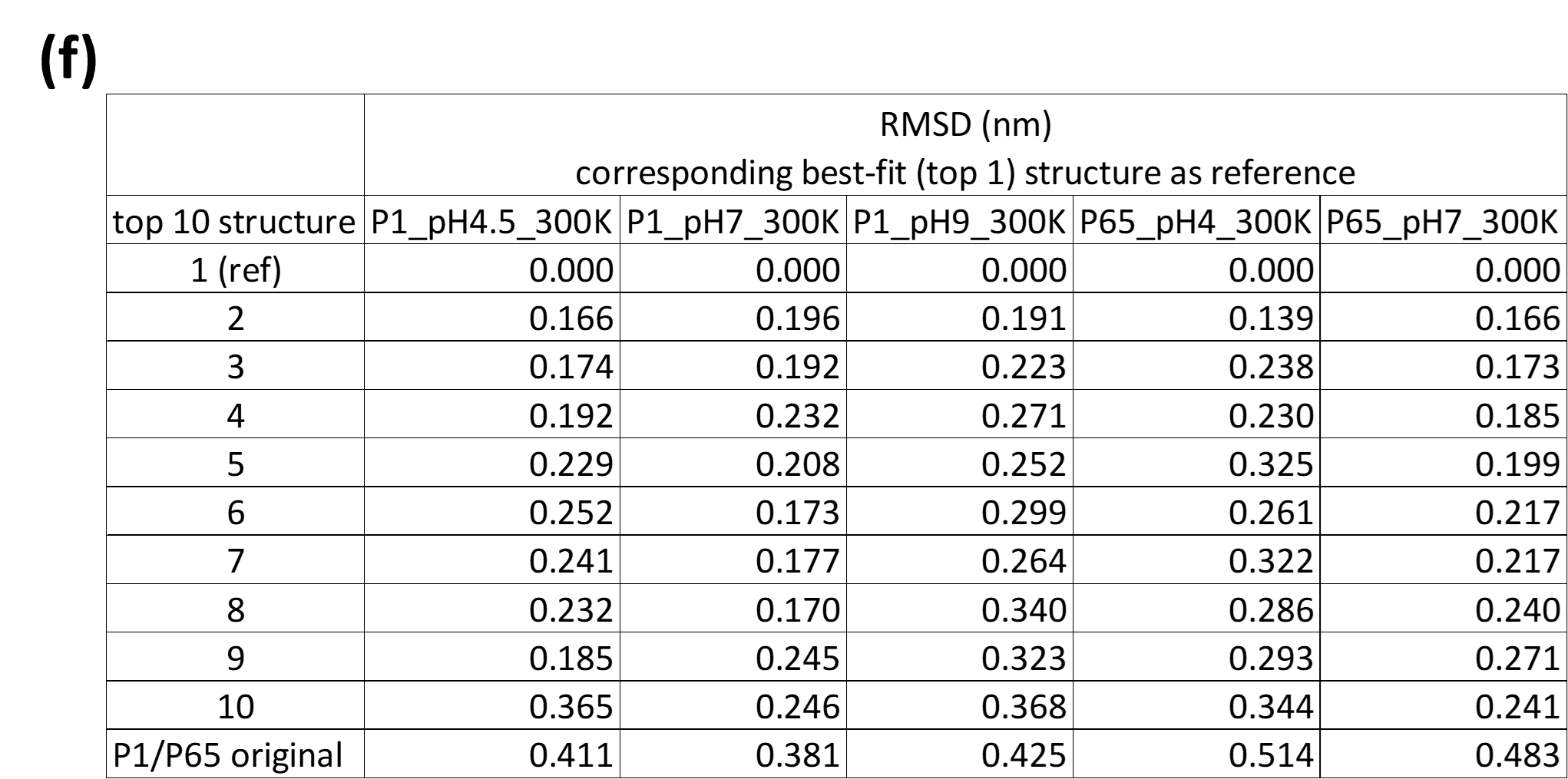


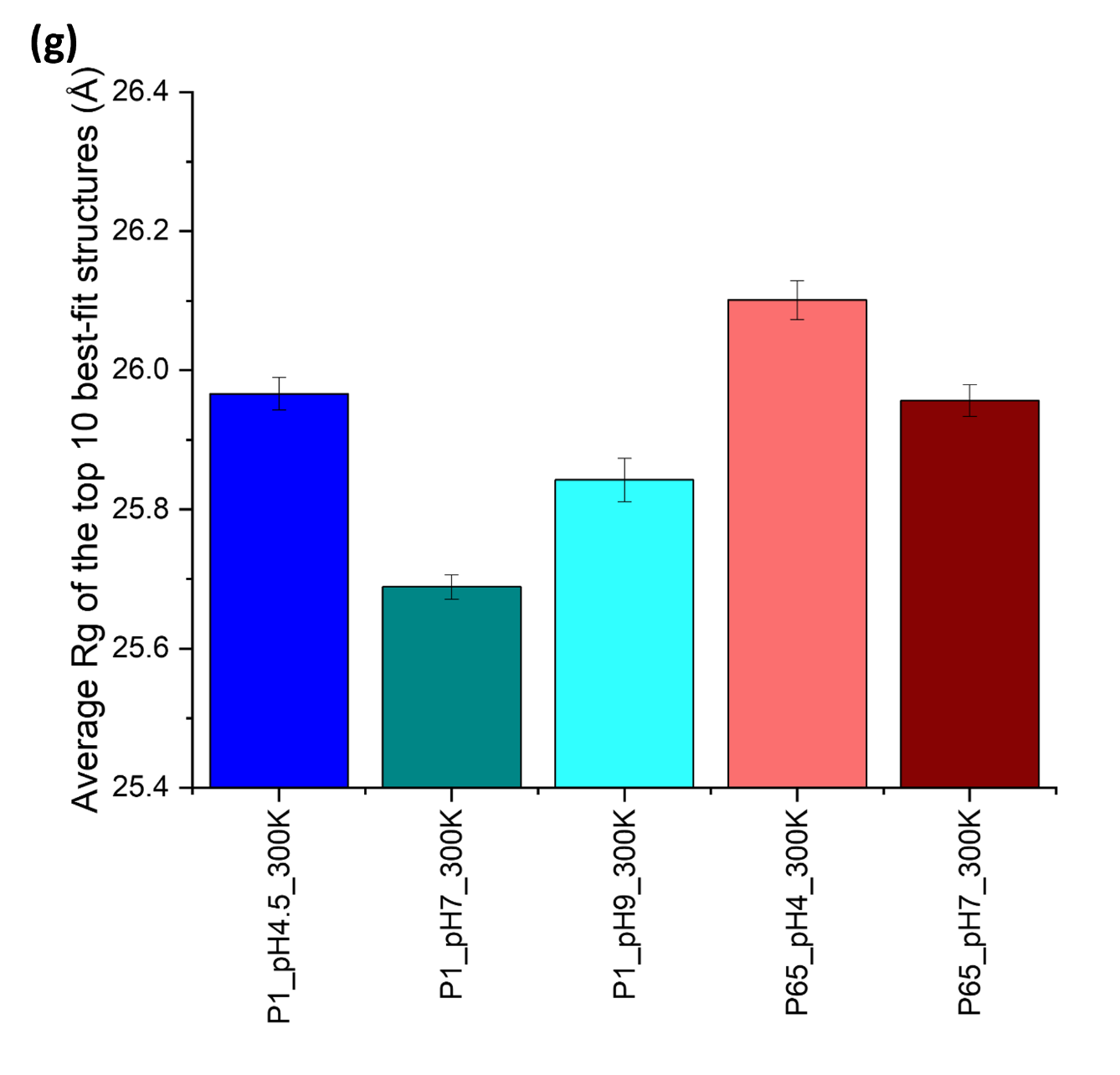


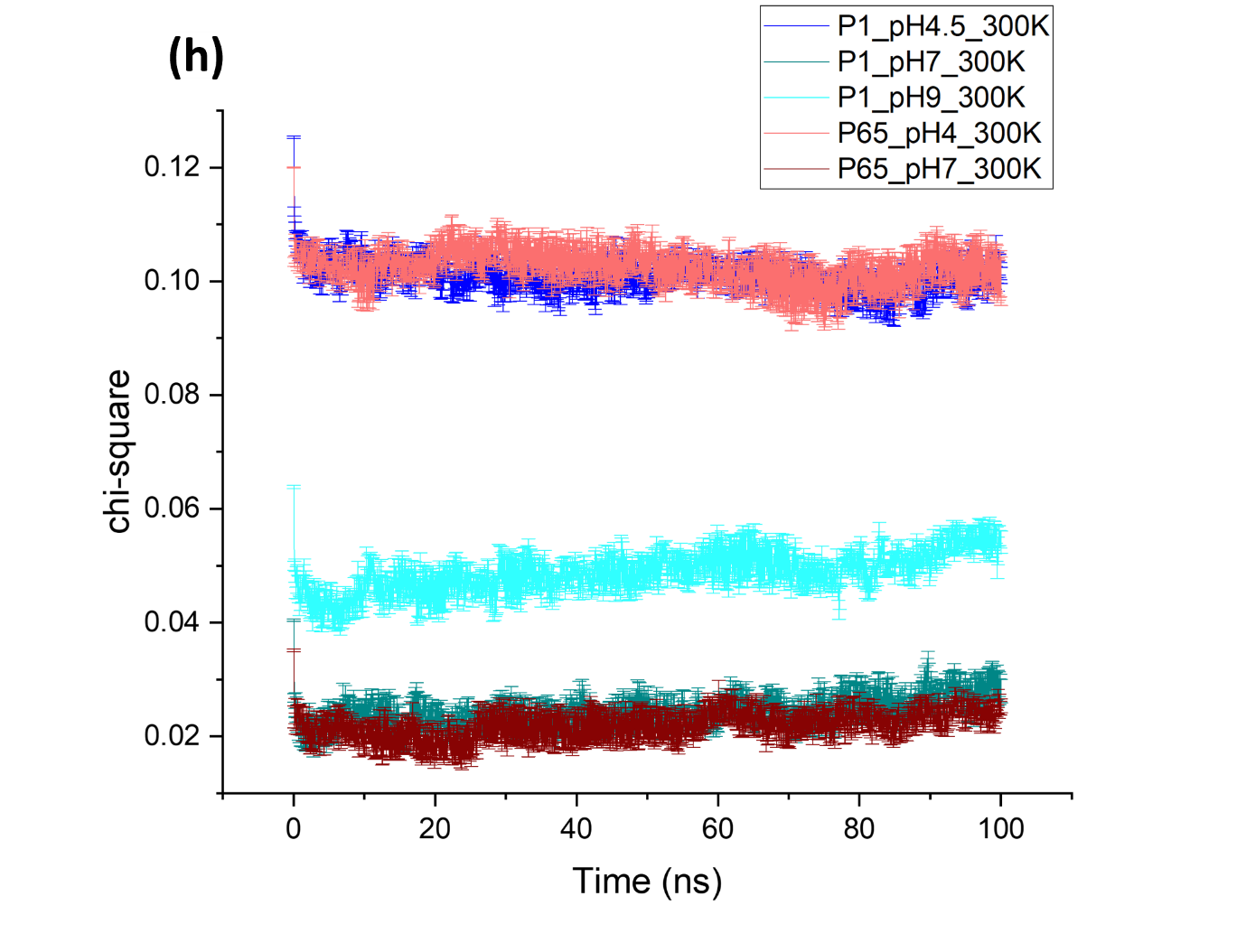


**Figure S3.** The summary for the SAXS curve fitting between the in silico and in vitro SAXS data. For each MD condition **(a-e),** 6006 (100 ns, 0.1 ns interval, 6 repeats) pair-wise SAXS curve fitting was performed as shown in Table 1, resulting 6006 dots plotted on the left figure for chi-square fitness and Rg. The top 10 best-fit frames (i.e. lowest chi-square) are coloured in black, with their structures aligned on the right. The details for the top 10 best-fit frames is tabulated in the middle table. The starting PDB used for MD is coloured in red in the chi-square vs Rg plot. The RMSD between the top 10 best-fit structures and P1/P6_5_ original structures is tabulated in **(f),** using the corresponding top 1 best-fit structures as the reference for RMSD calculations. The average Rg of the top 10 best-fit structures is shown in **(g)** with SEM as the error bar. The average chi-square of the SAXS fitting throughout the MD is shown in **(h)** with SEM as the error bar.
